# The InfAct proposal for a sustainable European health information infrastructure on population health: the Distributed Infrastructure on Population Health (DIPoH)

**DOI:** 10.1186/s13690-022-00844-z

**Published:** 2022-05-17

**Authors:** Rodrigo Sarmiento-Suárez, Alicia Padron-Monedero, Petronille Bogaert, Linda Abboud, Herman Van Oyen, Hanna Tolonen, Mariken Tijhuis, Stefanie Seeling, Romana Haneef, Metka Zaletel, Luigi Palmieri, Anne Gallay, Luís Velez Lapão, Paulo Nogueira, Thomas Ziese, Jakov Vukovic, André Beja, Miriam Saso, Isabel Noguer-Zambrano

**Affiliations:** 1 National School of Public Health. Instituto de Salud Carlos III, Avenida Monforte de Lemos 5, 28029 Madrid, Spain; 2 Medicine School, University of Applied and Environmental Sciences, Calle 222 #55-37, Bogota, Colombia; 3 Scientific Institute of Public Health, SciensanoRue Juliette, Wytsmanstraat 14, 1050 Brussels, Belgium; 4 Department of Public Health and Welfare, Finnish Institute for Health and Welfare (THL), Mannerheimintie 166, 00271 Helsinki, Finland; 5 National Institute for Public Health and the Environment RIVM, 3720 BA Bilthoven, Netherlands; 6 Department of Epidemiology and Health Monitoring, Robert Koch Institute, General-Pape-Straße 62-66, Berlin, Germany; 7 Santé Publique France, 12 Rue du Val d’Osne, Allée Vacassy, 94410 Saint-Maurice, France; 8 National Institute of Public Health, Trubarjeva 2, 1000 Ljubljana, Slovenia; 9 Department of Cardiovascular, Endocrine-Metabolic Diseases and Aging, Istituto Superiore Di Sanità (ISS), Via Giano della Bella, 34, 00161 Rome, Italy; 10 Instituto de Higiene E Medicina Tropical, Universidade NOVA de Lisboa, R. da Junqueira 100, 1349-008 Lisboa, Portugal; 11 Instituto de Medicina Preventiva E Saúde Pública, Faculdade de Medicina da Universidade de Lisboa, 1649-028 Lisboa, Portugal; 12 Croatian Institute of Public Health, Rockefeller str 7, 10000 Zagreb, Croatia

**Keywords:** Health Information, Health Information Systems, Non-Communicable Diseases, Population Health, Distributed Infrastructure on Population Health, InfAct

## Abstract

**Background:**

In Europe, data on population health is fragmented, difficult to access, project-based and prone to health information inequalities in terms of availability, accessibility and especially in quality between and within countries. This situation is further exacerbated and exposed by the recent COVID-19 pandemic. The Joint Action on Health Information (InfAct) that builds on previous works of the BRIDGE Health project, carried out collaborative action to set up a sustainable infrastructure for health information in the European Union (EU). The aim of this paper is to present InfAct’s proposal for a sustainable research infrastructure, the Distributed Infrastructure on Population Health (DIPoH), which includes the setup of a Health Information Portal on population health to be maintained beyond InfAct’s time span.

**Methods:**

The strategy for the proposal was based on three components: scientific initiatives and proposals to improve Health Information Systems (HIS), exploration of technical acceptability and feasibility, and finally obtaining high-level political support.. The technical exploration (Technical Dialogues—TD) was assumed by technical experts proposed by the countries, and political guidance was provided by the Assembly of Members (AoM), which gathered representatives from Ministries of Health and Science of EU/EEA countries. The results from the AoM and the TD were integrated in the sustainability plan compiling all the major outputs of InfAct.

**Results:**

The InfAct sustainability plan was organized in three main sections: a proposal of a new research infrastructure on population health (the DIPoH), new health information tools and innovative proposals for HIS, and a comprehensive capacity building programme. These activities were carried out in InfAct and are being further developed in the Population Health Information Research Infrastructure (PHIRI). PHIRI is a practical rollout of DIPoH facilitating and generating the best available evidence for research on health and wellbeing of populations as impacted by COVID-19.

**Conclusions:**

The sustainability plan received wide support from Member States and was recognized to have an added value at EU level. Nevertheless, there were several aspects which still need to be considered for the near future such as: (i) a commitment of stable financial and political support by Member States (MSs), (ii) the availability of resources at regional, national and European level to deal with innovations, and (iii) a more direct involvement from EU and international institutions such as the European Centre for Disease Prevention and Control (ECDC), the World Health Organization (WHO) and the Organisation for Economic Cooperation and Development OECD for providing support and sustainable contributions.

## Background

Health systems are one of the most important contributors to population health. However, demographic determinants of health such as population ageing, technological innovations in health care, and growing citizen expectations, among other factors, have increased financial constraints on health systems. On the other hand, increasing national health expenditures are expected to meet growing demands. Health information allows countries to develop population health-oriented research that increases the knowledge-base and underpins policy decision-making [1]. The term population health encompasses all relevant aspects of health information and combines the domains of health status, determinants of health and healthcare systems at individual and population level [2].

There is no single comprehensive EU health information system that allows policy oriented research or advice. The current EU health information and data landscape has issues of timeliness and usability [3]. Data on population health in the EU is fragmented, difficult to access, project-based and prone to health information inequalities in availability, accessibility and in quality between and within European countries [4]. As a consequence, comparability of the data is at stake. A clear governance of health information as the foundation of public health at EU level is lacking and differences in health information capacity in MS and associated countries are not systematically addressed [3]. Such landscape has deteriorated even further after the onset of the COVID-19 pandemic [5].

The Joint Action (JA) on Health Information (InfAct), which started in 2018, was built on previous initiatives that have been funded by the European Commission working towards the development of a sustainable infrastructure for health information. These initiatives include projects such as the European Core Health Indicators (ECHI) that since 1998 has been aimed at monitoring and comparing health outcomes between EU countries and therefore supporting policy-making processes [6]. Thereafter, the European Commission formulated its Third Health Programme 2014–2020 and one of the main objectives was to “contribute to innovative, efficient and sustainable health systems” [7]. This objective led to the project Bridging Information and Data Generation for Evidence-based Health policy and research (BRIDGE Health) in 2015 which prepared the transition towards a sustainable and integrated EU health information system for both public health and research purposes [1, 8]. The results of this project laid the foundation for the concept of a comprehensive health infrastructure, which was further developed in the InfAct project [9]. The mission of the InfAct was to build an EU health information system (EU HIS) infrastructure and strengthen its core elements to improve the use of health information data and expertise for a healthier Europe. To reach these, it would require establishing a sustainable research infrastructure to support the exchange of population health information, strengthen the European health information and knowledge base, as well as health information research capacities to reduce health information inequalities, and to support health information interoperability and innovative health information tools and data sources [10].

It is essential to ensure sustainability of the key actions to assure stability and functionality of them beyond the lifespan of InfAct. Many excellent initiatives in HIS and research fade out after their funding ends. This paper presents InfAct’s proposal for a sustainable research infrastructure, the Distributed Infrastructure on Population Health (DIPoH), which includes the setup of a Health Information Portal on population health to be maintained beyond InfAct’s time span.

## Methods

Under the framework of InfAct, the strategy to establish a sustainability plan for a EU health information system infrastructure was based on the following three components: 1) scientific initiatives and proposals to improve national and EU HIS; 2) exploration of technical acceptability and feasibility of those proposals, and 3) obtaining Member States’ high-level political support for them. Furthermore, to achieve a comparable and sustainable structure on population health across Europe, the health information inequalities of data quality at national level must be addressed through different initiatives such as capacity building and knowledge translation that facilitate the exchange of good practices between countries. All components were foreseen within the JA and gathered in a work package on sustainability and integration into policies.

Results from the InfAct activities were summarized and published in deliverables and fact sheets [11]. These documents covered topics such as the status of HIS in MS, health information capacity building activities, tools and methods for health information support, a proof of concept for a sustainable structure on health information, innovative methods for public health policy development and interoperability in public health. These outcomes and proposals were discussed during Technical Dialogues (TD) with national experts in health information appointed by Ministries of Health and national public health institutes [12, 13]. The TD facilitated the exchange of InfAct results with national counterparts, assessing their added value and the feasibility of integrating such outcomes and good practices at national and EU level. Finally, the Assembly of Members (AoM), which gathered high level representatives from the Ministries of Health and Science in countries provided political guidance for their acceptance [13]. The sustainability plan for a common EU health information system infrastructure was built on the integration of InfAct outcomes and activities, their technical feasibility and their political acceptance.

## Results

The InfAct sustainability plan for a European health information infrastructure was organized in three main sections: 1) A proposal for a new research infrastructure, 2) New health information tools and innovative proposals for HIS, and 3) A comprehensive capacity building proposal (Table 1). These sections are further addressed below.

**Table 1:** Main outcomes of the InfAct sustainability plan

Categories of the Sustainability Plan	Main InfAct Outcomes
Research Infrastructure Final Proposal	•Distributed Infrastructure on Population Health (DIPoH) •Proof of concept for a sustainable structure on health information •A sustainable network of networks •Health Information Portal
Health Information Tools and innovative proposals	•Health data collection methods and procedures •Cataloguing international health information networks, projects and indicator/datasets •A sustainable European Core Health Indicators (ECHI) shortlist •Guidance for health reports and recommendations for health reporting •Roadmap for innovative use of data sources •Best practices for innovative use of health information •Methodological guidelines for estimating health indicators using linked data and Machine Learning Techniques •A generic method case study and inspiring examples and Machine Learning Techniques •Use of non-health databases for health surveillance •Composite health indicator for monitoring non-communicable diseases (NCD) •Assessing and piloting interoperability
Capacity building	•A manual to carry out Health Information System (HIS) assessment in peer review format •Approaches to prioritizing health information at national level •Burden of disease workshops •Health information training program and roadmap for sustainability

### New infrastructure: the Distributed Infrastructure on Population Health (DIPoH)

The major outcome from the InfAct is a proposal to set up a sustainable infrastructure for population health information at the EU level to support availability of comparable, robust and policy-relevant health information on population health. For that purpose, a proposal for a Distributed Infrastructure on Population Health (DIPoH) has been prepared and submitted to the European Strategy Forum on Research Infrastructures (ESFRI) roadmap in 2020. The ESFRI was established in 2002 following a mandate by the EU Council to support research infrastructures across Europe aimed at facilitating scientific integration and knowledge translation in the region [14]. Acceptance on the ESFRI roadmap would provide an endorsement at EU level for the set-up of the infrastructure and would make DIPoH eligible for a more sustainable stream of financing.

DIPoH was designed on the basis of a federated infrastructure, respecting country and research ownership, EU-General Data Protection Regulation (GDPR) and following interoperability procedures. Its technical and scientific description was developed through a stakeholder consultation and consensus-driven approach in BRIDGE Health and further refined in InfAct [8, 9]. Based on this, the format has been found suitable and reliable to provide support towards the development and use of large-scale, integrated and sustainable data and information services for population health and health services research. The Research Infrastructure is expected to contribute to cataloguing, curating and integrating information and knowledge generated by a critical and growing mass of European researchers and their international networks. DIPoH will be able to strengthen the synergy in the EU by facilitating comparative research, efforts at data linkage, pan-European use of data, methods, expertise and results, and better involvement of national experts (NE). To support DIPoH, an integrated network of networks, composed by National Nodes (NN) and Research Networks (RN), will perform high-quality comparative research that will focus on regional, national and local health and health care issues, to support policy-making or health system improvement in a timely and effective way. A NN is an organisational entity, often linked to a national institution or governmental unit that functions as a national liaison and brings together relevant national stakeholders in the country in a systematic way, and RN is an active network of national and/or regional experts from several countries that perform comparative research in a specific health area. The foundation and building blocks of DIPoH, such as underlying concepts, users and services, governance, organisational elements, and costs and revenues, have been captured in a business plan. This plan will be fine-tuned over the next years.

An important part of the proposed DIPoH is the Health Information Portal (www.healthinformationportal.eu), a one-stop shop facilitating access to population health and health care data, information and expertise in the EU. It brings together currently scattered information to support their findability, accessibility, interoperability and re-use (FAIR). Basic functionalities of this portal have already been set up during InfAct and will be further developed under the framework of DIPoH. The NN function is to upload the information regularly and the potential users are researchers, policy makers and public health professionals [15].

In November 2020, a practical rollout of DIPoH in the area of COVID-19 has been implemented, the Population Health Information Research Infrastructure (PHIRI). PHIRI aims at facilitating and supporting open, interconnected, and data-driven research through the sharing of cross-country COVID-19 population health information and exchange of best practices [2]. In the COVID-19 crisis it has contributed to filling the gap of an urgently needed rapid exchange of data and information between countries and linking with other initiatives on health information at national and European level. PHIRI will further implement activities from InfAct such as expanding the reach of the Health Information Portal to include also population health information, data and expertise related to COVID-19, boosting innovation in the area of population health, strengthening European public health capacities and supporting translation of research to policy.

### Health information tools and innovative proposals

DIPoH aims to support researchers by ensuring their research is FAIR (findable, accessible, interoperable, re-usable) and to create stronger research networks, providing a data catalogue on health status, health determinants and health care data, and methodologies used. It also aims to invest in health information development and innovative methods for population health research to support researchers using pan-European data in a distributed way.

In InfAct, the following health information tools and innovations were developed, piloted and made available to the Health Information Portal, for use in DIPoH:

- A roadmap of innovative use of data sources, identifying best practices for using health information and methodological guidelines, which could systematically guide EU MS and associated countries (AC) for using linked data and machine learning techniques to estimate health indicators for public health research [16–18].
- The development of new composite indicators to improve the monitoring of NCD: the “En-risk” an interactive application tool, which uses European non-health databases of industrial pollution and its association with mortality for public health surveillance, and the ratio between hospital admissions and deaths to explore the geographical variability of morbidity and mortality in a country [11].
- InfAct has assessed interoperability by in-depth interviews with key opinion leaders and experts to better understand the enablers and the barriers to the cross-border linkage and sharing of health data through the four interoperability layers (legal, organisational, semantic and technical) and has piloted interoperability through three case studies to capture different requirements in the development of a distributed infrastructure on population health research. Most key experts emphasized legal and semantic interoperability layer as a main barrier, while technical interoperability was no longer seen as a barrier unless practicing physicians and patients are involved [11]. Other barriers emphasized by these experts were lack of funding, differences in health data in countries with decentralized governments and different interpretations of the GDPR that varied between countries, between different regions of a country and between different institutions. On the other hand, the pilot exercise showed that data hubs are well prepared to run different analysis with shared scripts of the proposed federated model [19].
- Tools to improve methods and procedures for health data collection, a guidance for health reports with recommendations for health reporting, and suggestions for an update and sustainable governance structure for the ECHI shortlist [11, 20, 21]. The list provides a snapshot overview of European public health. It is the result of consecutive EU-wide projects representing a collective MSs effort, implemented in 2012. InfAct provides recommendations for a governance structure and formal procedures for regularly updating the ECHI shortlist [6, 11].

### Capacity building

DIPoH is committed to increase capacity building in EU by providing training that supports both data users and producers, to tackle health information inequalities across Europe. In the framework of InfAct, several capacity building activities were developed and piloted: (i) HIS assessments in peer review format in nine countries, identifying strengths and weaknesses in the national HIS under assessment and providing recommendations to improve the assessed HIS [22]. The methodology applied for these peer assessments is derived from the methodology developed and piloted by WHO Regional Office for Europe in the framework of the WHO European Health Information Initiative (EHII), the “Support tool to assess health information systems and develop and strengthen health information strategies” [23]. In InfAct, this methodology has been adapted to make it suitable for peer review assessments. An important distinction is that InfAct assessments were initiated and executed at the level of health information institutions and experts [24]. The WHO support tool has been revised and received the feedback from the InfAct peer review assessment experience for future assessments to be carried out in the region [25]. (ii) A flagship programme of training has been designed to address existing inequalities and to improve the MSs capacities in population health data collection, monitoring, data analysis and knowledge translation. This programme was implemented as a five-day course, addressing from data collection tools to GDPR and policy translation, engaging 22 participants across Europe and 19 international lectures [26]. The European Health Information Training Programme (EHITP) was conceptualized as an umbrella for all current and future training activities in Europe, targeting professionals working in public health and health information at national or international level. (iii) A two-round Delphi survey on existing approaches to health information development and prioritization methods was conducted, and recommendations were drafted on the topics that a guidance document on prioritization should include [27, 28]. (iv) The burden of disease (BoD) methods quantify the comparative magnitude of health loss due to disease, injury and risk factors. These methods can add value to existing approaches on public health. Three workshops on BoD methodology have been organized to support countries in conducting their own national BoD study. As a result, a set of recommendations has been developed to plan a national BoD study [29, 30].

## Discussion

Main results from InfAct are the development and implementation of dedicated services of the Distributed Infrastructure on Population Health (DIPoH), providing HI innovative proposals, capacity building on HI, research results, managerial and surveillance databases and a wide offer and access to different population health data, available and accessible for decision-makers. According to the technical assessment from the TD, all countries coincided in the added value of InfAct outcomes, articulated around DIPoH to enhance population health research across Europe, to eventually improve health and wellbeing of the citizens in the region. The main concern raised during the TD was related to the European General Data Protection Regulation (GDPR) because it might affect interoperability and restrictions in comparable data, due to differences in the interpretation and implementation of the GDPR across countries. Therefore, it was mentioned that InfAct should explore further options to perform data linkage, sharing, management and reporting in the near future. In addition, an EU-consensus guideline for data anonymization is urgently needed [12, 13].

Political and institutional support for the DIPoH initiative has been confirmed by providing letters of political support or signing an institutional Memorandum of Understanding. So far 14 countries have made that support explicit. Among the barriers for a wider political support, countries still have some concerns about financial contribution, and organizational and managerial aspects. Also some countries would support a mandate extension of the ECDC rather than a new research infrastructure. However, such extension is not a possibility in the near future because of its current focus on infectious diseases, especially now with the COVID_19 pandemic, and has no expertise on Non Communicable diseases [9]. In the long term, an extension of the ECDC would be of mutual benefit for both infrastructures as DIPoH would cover the activities not included in this potential new mandate. On the other hand, these countries that preferred the ECDC extension have become more acknowledged of the added value of DIPoH.

### Limitations

Although the sustainability plan has found broad support and its added value has been widely acknowledged, several aspects remain key issues to be considered for the near future. Firstly, MS/AC should assure the technical, political and financial stability in funding and resources, therefore stronger and more extended support from MS/AC would be necessary to achieve DIPoH’s goals. The current implementation of PHIRI as a rollout of DIPoH might have a snowballing effect as the countries that are still hesitating to support this infrastructure can assess how DIPoH operates in practice. Secondly, it will require both technological and human resources at regional, national and European level to develop innovative proposals aimed at improving health information. Thirdly, the whole sustainability plan requires further involvement from EU and international institutions as ECDC, WHO, OECD and also existing research networks such as Euro-Peristat. The support of these organizations and networks is essential to a fully functioning one-stop shop for EU health information research because they are important data providers and users of the metadata catalogues and tools of DIPoH. Lastly, although COVID-19 has helped to stress the need for an infrastructure such as DIPoH and has spurred the creation and funding of PHIRI, the onset of future public health emergencies or similar exceptional circumstances may thwart the continuous financial support from MS/AC, and generate different pace and interest. In spite of that, we consider DIPoH to be a flexible instrument that has already proved itself useful in new public health emergencies as during the current COVID-19 crisis. For instance, setting up a cross-country exchange between experts from national public health institutions that participated in InfAct has allowed many countries to obtain up-to-date evidence-based information on COVID-19 management for supporting public health interventions and health policy decision making during the pandemic [31].

## Conclusion

Improving the health of the European citizens and increasing the efficiency of health care systems and policy decisions would be InfAct’s added value. A strong EU health information system is needed to tackle the public health challenges EU MS/AC are facing. This requires a solid and sustainable infrastructure to support health research, which provides a robust evidence base for policy decisions, allocation of health care resources and targeting of health care services. InfAct has paved the way for this infrastructure by preparing the Distributed Infrastructure on Population Health (DIPoH). Through its one-stop shop, DIPoH facilitates high-quality, innovative research supporting evidence-informed policy decisions in the full domain of public health and care, which will help the health policy and health research communities to have access to new knowledge and means to translate this knowledge into action.

## Data Availability

All data and information generated or analysed during this work are available from InfAct and at the following links: www.inf-act.eu https://www.inf-act.eu/assembly-members

## Abbreviations

AoM: Assembly of Members
BoD: Burden of Disease
DIPoH: Distributed Infrastructure on Population Health
ECDC: European Centre for Disease Prevention and Control
EHITP: European Health Information Training Programme
ESFRI: European Strategy Forum on Research Infrastructures
EU-EEA: European Union European Economic Area
GDPR: General Data Protection Regulations
HIS: Health Information Systems
HSP: Healthcare Systems Performance
InfAct: Information for Action
JA: Joint Action
MS/AC: Member States and Associated Countries
NCD: Non-communicable Diseases
PHIRI: Population Health Information Research Infrastructure
TD: Technical Dialogues
